# Take a stand on your decisions, or take a sit: posture does not affect risk preferences in an economic task

**DOI:** 10.7717/peerj.475

**Published:** 2014-07-17

**Authors:** Megan K. O’Brien, Alaa A. Ahmed

**Affiliations:** 1 Department of Mechanical Engineering, University of Colorado Boulder, Boulder, CO, United States; 2 Department of Integrative Physiology, University of Colorado Boulder, Boulder, CO, United States

**Keywords:** Decision-making, Neuroeconomics, Economic lottery, Risk-sensitivity, Posture, Threat, Skin conductance, Prospect theory

## Abstract

Physiological and emotional states can affect our decision-making processes, even when these states are seemingly insignificant to the decision at hand. We examined whether posture and postural threat affect decisions in a non-related economic domain. Healthy young adults made a series of choices between economic lotteries in various conditions, including changes in body posture (sitting vs. standing) and changes in elevation (ground level vs. atop a 0.8-meter-high platform). We compared three metrics between conditions to assess changes in risk-sensitivity: frequency of risky choices, and parameter fits of both utility and probability weighting parameters using cumulative prospect theory. We also measured skin conductance level to evaluate physiological response to the postural threat. Our results demonstrate that body posture does not significantly affect decision making. Secondly, despite increased skin conductance level, economic risk-sensitivity was unaffected by increased threat. Our findings indicate that economic choices are fairly robust to the physiological and emotional changes that result from posture or postural threat.

## Introduction

Have you ever wondered whether you were in the right frame of mind to make a decision? Converging evidence suggests that physiological and emotional states affect decision making, even when these states are not particularly salient to the decision task. For example, a recent study demonstrated that metabolic state can alter risk-sensitivity in an unrelated economic decision-making task, suggesting similar neurobiological correlations for the representation of value and uncertainty across task domains (Symmonds et al., 2010). Another group found that action planning can influence our perceptions (Witt & Brockmole, 2012). When holding a gun, subjects were more likely to perceive objects held by others as guns, and they were more likely to exhibit threatening behavior, such as raising the gun to a shooting posture. When holding neutral objects, such as a ball or a shoe, subjects were more likely to identify objects held by others as neutral objects rather than guns. This outcome appears to support a theory of event encoding, where action planning biases perception (i.e., planning an action involving a gun results in a bias to identify other objects as guns) because action-based and perceptual representations involve shared neural processes. Indeed, there could be many subtle changes in our bodies or environment that contribute to choices we make under risk.

Previous studies have found physical and neurobiological implications of adopting certain body postures. For instance, standing is less comfortable than sitting, causing more fatigue and particular discomfort in the feet and lower limbs over prolonged period of time (i.e., 90 min) (Chester, Rys & Konz, 2002; Drury et al., 2008). Standing is more biomechanically unstable than sitting and is more likely to result in a fall. Standing is also more cognitively loading than sitting, requiring greater attentional demands to maintain the posture (Teasdale et al., 1993; Lajoie et al., 1993; Lajoie et al., 1996). High-power poses—in which the body is open and expansive—increase testosterone and decrease cortisol levels, whereas low-power poses—in which the body is closed and contracted—have the opposite effect (Carney, Cuddy & Yap, 2010). Posture may also influence our perception, performance, and decision-making processes, potentially as a result of the accompanying physiological changes. More comfortable postures can enhance performance in memory tasks (Lipnicki & Byrne, 2005; Yardley et al., 2005), while less comfortable positions can improve reaction time (Vercruyssen & Simonton, 1994). Adopting high-power poses for as little as one minute leads to increased feelings of power as well as risk-seeking behavior in a gambling task (Carney, Cuddy & Yap, 2010), congruous with the neuroendocrine profiles that accompany such poses. However, this risk-seeking behavior was found for monetary losses and involved a single lottery, rather than examining a range of monetary amounts and probabilities. It is unclear whether more neutral, commonplace postures such as sitting or standing would influence risk-sensitivity for monetary gains. In an earlier study of risk-sensitivity for a motor task, we found that subjects were more risk-seeking in a standing whole-body movement than in a seated arm-reaching movement (O’Brien & Ahmed, 2013). Was this difference in risk-sensitivity due to the types of movement, or simply because of the sitting and standing postures? In the present study, we sought to differentiate possible changes in risk-sensitivity due to the postures themselves using a non-motor task.

Postural threat has also been shown to alter our behavior, particularly in the movement domain. There are multiple examples of altered postural control in the context of minimal increases in postural threat: forward vs. backward leaning (Manista & Ahmed, 2012), standing with a narrow vs. a wide stance width (Pienciak-Siewert, Barletta & Ahmed, 2014), and standing on a reduced base of support (Huang & Ahmed, 2011). Modest changes in elevation also induce marked changes in motor control and physiological arousal, indicating greater anxiety (Ashcroft et al., 1991; Brown et al., 2002; McKenzie & Brown, 2004; Brown et al., 2006; Brown, Polych & Doan, 2006). When asked to walk or simply stand on an elevated platform, both young and old adults reduce the velocity and extent of their movements (Adkin et al., 2002; Carpenter et al., 2006; Adkin et al., 2008; Davis et al., 2009; Lamarche et al., 2009). During quiet standing, postural control variables are scaled to surface height, with center of pressure (COP) displacements decreasing in amplitude and increasing in frequency at higher elevations, up to 1.6 m but as low as 0.81 m (Carpenter, Frank & Silcher, 1999; Adkin et al., 2000; Carpenter et al., 2001). At a surface height of 0.81 m, individuals adopt a stiffening strategy during quiet standing, increasing activity in anterior leg muscles and shifting their COP away from the surface edge (Carpenter et al., 2001). Typically, these changes are attributed to a fear of falling that affects the action selection process of the central nervous system (CNS). If changes in movement tasks on elevated platforms are a result of the feelings of threat experienced while standing on the platform, then it is feasible that these emotions will influence risk-sensitive behavior in non-motor tasks as well.

Together, these findings compel us to further examine the effects of physiological and emotional state on decision making. Here we specifically studied the influence of body posture and postural threat on economic decisions. Subjects performed a two-alternative forced choice lottery task under various conditions. We compared their risk preferences across two body postures (sitting vs. standing) and two levels of postural threat in the form of elevation (ground level vs. atop a 0.8 m platform). We expected that the more uncomfortable body posture (standing) and higher postural threat (atop the platform), would lead to more risk-averse choices during economic decision making. This conjecture is predominantly based on preceding investigations of the role of affect in judgment and decision making, which suggest that our actions are often based on avoiding negative emotions (Slovic, 1987; Finucane et al., 2000; Loewenstein et al., 2001; Slovic et al., 2002; Loewenstein & Lerner, 2003; Slovic et al., 2004). Because standing induces relative discomfort, biomechanical instability, and attentional demands, and because elevation magnifies a fear of falling, we anticipated that subjects’ desire to avoid such negative states would contribute to a desire to avoid risk that would carry over to the economic domain. If risk-sensitivity were altered by even subtle changes in feelings of discomfort or threat, this would further assert that consideration of state is fundamental to the ability to mechanistically predict decisions across domains. Conversely, similar risk-sensitivity between conditions would indicate that economic choices bear a level of resistance to physiological and emotional changes.

## Materials and Methods

### Ethics statement

All subjects provided written informed consent before participation. The experimental protocol (12-0458) was approved by the Institutional Review Board of the University of Colorado Boulder in accordance with federal regulations, university policies, and ethical standards regarding human subject research.

### Experimental protocol

Thirteen healthy subjects (8 females, 5 males; mean age, 23.1 ± 2.2 years) participated in this experiment. These subjects were part of a broader study examining the influence of threat on non-motor and motor tasks. Subjects made choices in an economic lottery series in four conditions: sitting at low elevation (SIT Low), standing at low elevation (STAND Low), sitting at high elevation (SIT High), and standing at high elevation (STAND High). Throughout a series, subjects were asked to choose between two lotteries, where each lottery has a different monetary reward and probability of winning that reward.

For the Low conditions, subjects either sat in a chair or stood at the edge of a forceplate (AMTI Dual-Top AccuSway, which is 4.5 cm in height). For the High conditions, subjects sat in the same chair or stood on the same forceplate at the edge of an elevated platform, 0.8 m off the ground. The height of this platform was equivalent to that of an average table or desk, and it is approximately the average height at which young adults perceive they would not be able to use a step down strategy to descend from an elevated surface (Brown & Frank, 1997). When standing in either elevation condition, subjects were secured in a harness and fall protection system that could arrest a fall before the subjects’ knees touched the platform. However, to maintain perceptions of postural threat in the presence of this added safety, there was enough slack to the harness to allow subjects to move without restraint, and they were not allowed to explore the competence of the fall protection system before testing.

Subjects performed the SIT and STAND lottery tasks in a randomized order at each elevation, counterbalanced across the two tasks. They completed both choice tasks at Low elevation before performing them at High elevation. Previously, it was shown that increasing elevation results in more pronounced changes to postural control variables than decreasing elevation (Adkin et al., 2000). In presenting the Low elevation condition first, we intended to capitalize on these order effects to maximize changes in postural control due to threat and, thus, to maximize potential changes in the action selection process.

Lotteries were displayed on a computer monitor in front of the subject. In the testing phase of the experiment, subjects simply chose between pairs of lotteries using a two-button remote. Subjects performed 72 choice trials for each condition, where every choice completed a single trial. Lottery information for each trial was shown for 4 s; the lotteries then disappeared and subjects were given 2 s to select their preferred lottery. There were no failed trials; all subjects provided a response to every trial.

After completing the four conditions, subjects participated in a realization of choices phase. We randomly selected one trial from each condition, and the subject “played” their choice on that trial for real money. We used a random number generator to determine whether a subject won the monetary reward presented in that choice. Subjects were aware of the random selection of trials to be played in order to ensure their decisions were representative of what they would do in a real-life scenario.

### Lottery design

We adapted a lottery series design from Wu, Delgado & Maloney (2011). Subjects chose between two lotteries (A and B), each of which had a different monetary reward ($*y* and $*z*) and probability of winning that reward (*p* and *q*). We formulated these lotteries as A($*y*, *p*) and B($*z*, *q*). For every trial, there was one “safer” lottery and one “riskier” lottery, which were classified based on the variance of each lottery. The lottery with a higher variance was considered the riskier option.

(1) \begin{eqnarray*} \begin{array}{l} \displaystyle \mathrm{V ar}[A]=p{y}^{2}(1-p)\\ \displaystyle \mathrm{V ar}[B]=q{z}^{2}(1-q). \end{array} \end{eqnarray*}

**Figure 1:**
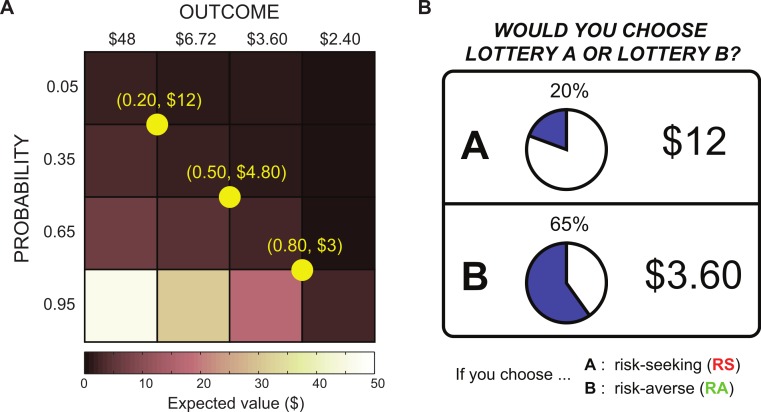
Lottery design. (A) Lotteries were constructed using a 4 × 4 outcome-probability matrix, where each block is paired with each reference lottery (shown in yellow). (B) Sample lottery presentation. Subjects were asked to choose between two economic lotteries, with differing monetary rewards and probabilities of winning those rewards.

Lottery pairs were presented in three blocks of 24 trials, for a total of 72 trials per task. Each lottery pair consisted of a reference lottery and a varying lottery. The reference lottery was fixed within a block, whereas the varying lottery changed from trial to trial. We used a 4 × 4 outcome-probability matrix to construct the lottery pairs, as shown in Fig. 1A. The reference lotteries had the same expected value. For the varying lottery, there were four possible monetary outcomes ranging from $2.40 to $48, and there were four possible probabilities ranging from 0.05 to 0.95. The diagonal elements of the matrix had nearly the same expected value and were shown three times per block, while the remaining off-diagonal elements were shown once per block. We randomized the order of the blocks as well as the order of the varying lotteries for each subject and task. An example lottery pair is shown in Fig. 1B. Subjects were explicitly shown the rewards and probabilities for each lottery, but they were not told which lottery was safer or riskier on any given trial.

### Measures of risk-sensitivity

One metric we used to compare risk-sensitivity between conditions was the frequency of risky choices (fR) in each task. We computed fR by comparing how many times a subject chose the riskier lottery over the safer lottery to the total number of trials in a task. Although this metric does not provide information about risk preferences on individual trials, it provides global view of risk-seeking (or risk-averse) behavior that we can compare across conditions.

We also employed cumulative prospect theory (CPT) to estimate subject-specific distortions in the utilities and probabilities associated with our lotteries. In CPT, risk-sensitivity can be explained by either a distortion in the (1) utility/value function or (2) probability weighting function (Tversky & Kahneman, 1992). Utility refers to the subjective valuation of an outcome (such as money), and a utility function describes how that valuation changes across outcomes. For instance, people tend to perceive the difference between $5 and $10 as more meaningful than the difference between $105 and $110, even though the objective difference is $5 in both cases. This is an example of diminishing sensitivity to increasing outcomes and can be captured by modeling utility with a power function. Probability weighting relates the likeliness of an outcome to the desirability of that outcome. Empirical evidence has shown that individuals weight probabilities nonlinearly, usually overweighting small probabilities (unlikely events) and underweighting large probabilities (likely events).

Under the formalization of CPT, we used the following value function, *v*(*O*), and Prelec’s probability weighting function, *w*(*P*):

(2a) \begin{eqnarray*} \displaystyle v(O)={O}^{\alpha },\quad O\geq 0&&\displaystyle \end{eqnarray*}

(2b) \begin{eqnarray*} \displaystyle w(P)=\mathrm{exp}[-(-\ln (P))^{\gamma }],\quad 0\lt P\lt 1.&&\displaystyle \end{eqnarray*}

The relevant parameters for utility and probability weightings are *α* and *γ*, respectively. Distortions in utility and probability (*α*, *γ* ≠ 1) characterize risk-sensitive behavior, with *α* < 1 and *γ* < 1 indicative of risk-aversion and underweighting large probabilities, respectively. Conversely, *α* > 1 and *γ* > 1 are indicative of risk-seeking behavior and overweighting large probabilities, respectively.

Then, the cumulative prospects of the two lotteries, A($*y*, *p*) and B($*z*, *q*), are:

(3) \begin{eqnarray*} \begin{array}{l} \displaystyle {\psi }_{\mathrm{A}}=v(y)w(p)\\ \displaystyle {\psi }_{\mathrm{B}}=v(z)w(q). \end{array} \end{eqnarray*}

We used a logistic choice function with constant noise (Stott, 2006; Chib et al., 2012), so that the probability that a subject chooses lottery A is given by:

(4) \begin{eqnarray*} {P}_{\mathrm{A}}=\frac{1}{\mathrm{1 + exp}[-k({\psi }_{\mathrm{A}}-{\psi }_{\mathrm{B}})]}, \end{eqnarray*}

where *k* is a parameter that accounts for stochasticity in a subject’s choices. A stochasticity parameter *k* = 0 characterizes random choice.

We used maximum likelihood estimation to estimate subject-specific distortions in utility and probability for each task. The procedure for fitting these CPT parameters is as follows: on the *i*th trial, a subject makes a choice *r_i_*. Let *r_i_* = 1 denote choosing lottery A, and let *r_i_* = 0 denote choosing lottery B. A maximum likelihood estimation of the parameters (*α*, *γ*, *k*) is one that maximizes a likelihood function over *n* trials, which we write as:

(5) \begin{eqnarray*} L(\alpha ,\gamma ,k)=\prod _{i=1}^{n}P_{\mathrm{A}}^{{r}_{i}}(1-{P}_{\mathrm{A}})^{{r}_{i}}. \end{eqnarray*}

We used MATLAB’s *fminsearch* function with multiple starting conditions to minimize the negative value of this likelihood function and estimate each subject’s parameters.

### Skin conductance

Skin conductance measurements are often used as an indicator of anxiety, affective response, and emotional arousal. We measured changes in skin conductance throughout this experiment using the BIOPAC MP35 acquisition hardware, collecting data at 1,000 Hz. Disposable electrodes were placed on the subject’s left hand, on the distal phalanx of the index and middle fingers. Skin conductance level (SCL) for each subject was calculated as a percent increase over a baseline condition, during which subjects sat quietly for 5 min. SCL data is available for 12 of the 13 subjects; one subject’s SCL data is not presented due to a calibration error.

### Statistics

We used paired *t*-tests to compare SCL between the sitting and standing postures and low and high elevations. We performed a two-way repeated-measures analysis of variance (ANOVA) to determine whether there were effects of body position or elevation on our first measure of risk-sensitivity, fR. We used paired *t*-tests to examine potential differences in fR, as well as Fisher’s exact test to compare the distribution of subjects with *α* and *γ* values greater than 1.0 between conditions. Permutation testing was employed to further compare CPT parameter fits between conditions without making assumptions about the underlying distribution of the samples. For all statistical tests, the significance level was set to 5%.

## Results and Discussion

### Overview

We found no significant differences in risk-sensitivity between any conditions. Subjects chose riskier lotteries as frequently when sitting as they did when standing, and this frequency did not change between low and high elevation. Similarly, we did not see substantial changes in the parameter fits for utility and probability weighting between conditions.

### Skin conductance

**Figure 2:**
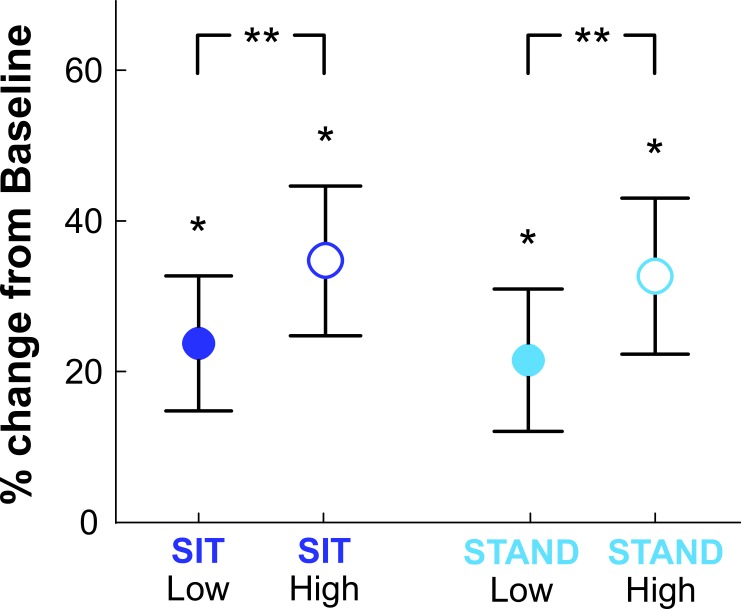
Skin conductance. Skin conductance levels (SCL) for all conditions relative to Baseline (quiet sitting). SCL in all conditions was significantly higher than at Baseline (^∗^*p* < 0.004), and SCL at the High elevation was significantly higher than at the Low elevation (^∗∗^*p* < 0.002). There was no difference in SCL between sitting and standing conditions at either elevation.

Mean SCL for the Low and High elevation conditions are given in Fig. 2. For each condition, SCL was significantly higher than at Baseline (*p* < 0.004). There was no difference in SCL between SIT and STAND at either elevation (Low: *p* = 0.89; High: *p* = 0.96), though SCL for conditions at High elevation were significantly higher than those at Low elevation (*p* < 0.002). These measurements suggest that subjects did indeed have a physiological response to elevation, but not to body posture.

### Frequency of risky choices

**Figure 3:**
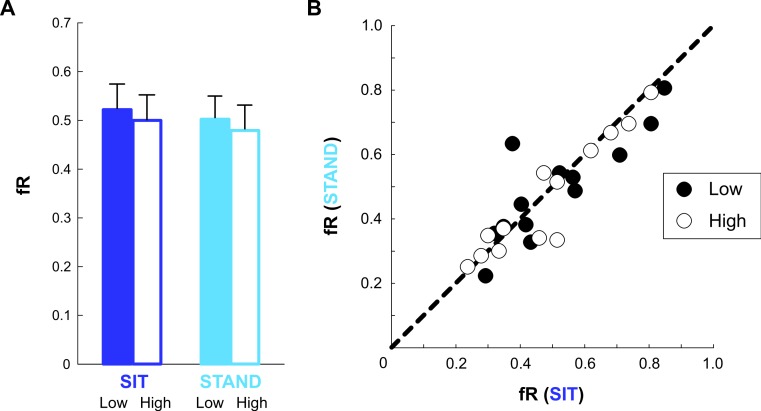
Frequency of risky choices. (A) Mean frequency of risky choices (fR) for SIT and STAND at Low elevation (filled bars) and at High elevation (outlined bars). (B) Each subject’s fR in the SIT condition compared with that in the STAND condition, at Low elevation (filled circles) and at High elevation (outlined circles). A data point on the line of unity indicates that the subject chose the same number of risky lotteries in both body postures.

Subjects chose the riskier lottery a comparable number of times regardless of condition. There were no significant differences in average fR between SIT and STAND at either elevation, and fR was similar between the Low and High elevations (Fig. 3A). This figure illustrates a slight, though consistent, trend to choose the risky lotteries less often under higher postural threat, with mean (± SEM) fR values of 0.51 (0.05) for SIT Low, 0.49 (0.05) for STAND Low, 0.48 (0.05) for SIT High, and 0.46 (0.05) for STAND High. However, paired *t*-tests between do not reveal significant differences in fR between any conditions. Figure 3B illustrates that individual fR values in SIT were nearly equal to those in STAND at both Low and High elevations. A repeated-measures ANOVA did not reveal effects of body posture (*F* = 0.11, *p* = 0.74), elevation (*F* = 0.26, *p* = 0.61), or an interaction between these factors (*F* = 0.0011, *p* = 0.97).

### CPT parameter fits

**Figure 4:**
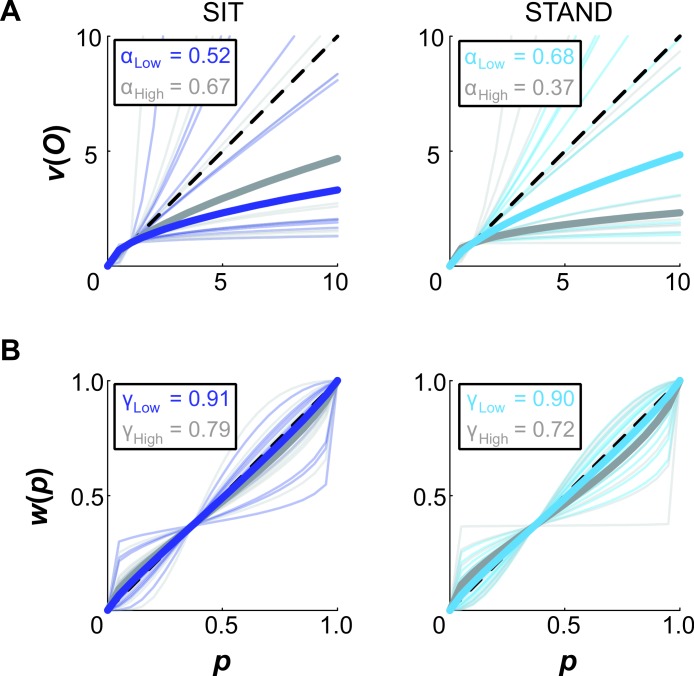
CPT curves. Cumulative prospect theory (CPT) model fits for (A) utility and (B) probability weighting in the SIT and STAND conditions. Thick lines indicate the median curves for Low elevation (colored) and High elevation (gray); thin lines correspond to fits for individual subjects.

**Figure 5:**
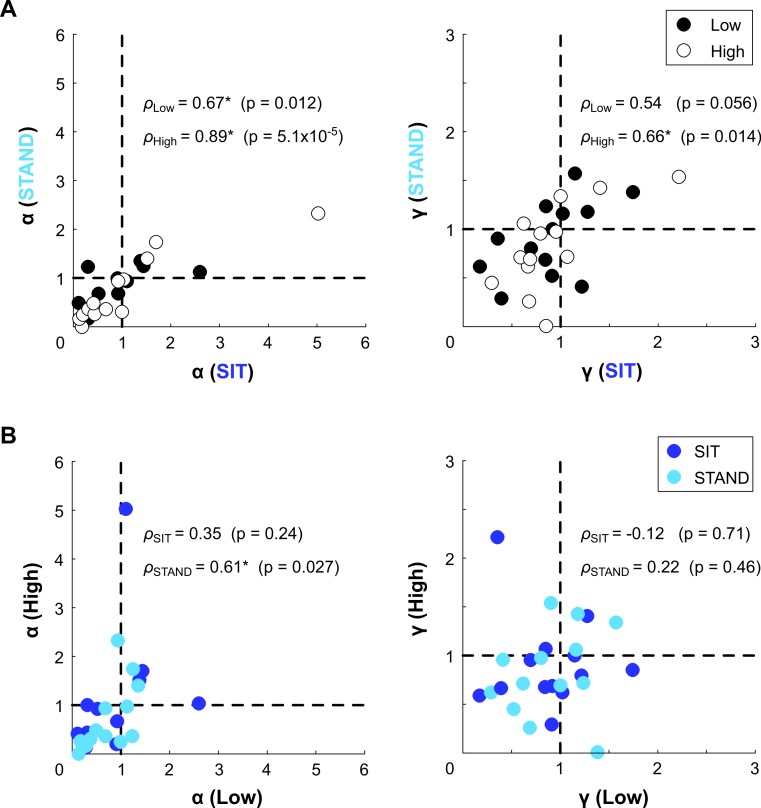
Individual CPT fits. Individual *α* and *γ* fits compared between conditions, including (A) body posture (SIT vs. STAND) and (B) elevation (Low vs. High). Risk preferences in utility were fairly consistent between conditions, as indicated by most *α* values lying within in the first and third quadrants of the plot. Probability weighting appears more idiosyncratic, with an increased number of values located in the second and fourth quadrants for *γ*. Correlation coefficients and their significance are also reported (^∗^*p* < 0.05).

Median parameter fits and 95% confidence intervals are given in Table 1. For both SIT and STAND, these median fits suggest risk-averse behavior in utility and a slight tendency to underweight large probabilities (Fig. 4). These trends hold for both elevations. A comparison of individual subjects’ CPT parameters between conditions is illustrated in Fig. 5. In these plots, if an individual’s general risk preferences did not change between the conditions of interest, we would expect data points to fall in the first quadrant (indicating consistent risk-seeking behavior in *α* and consistent overweighting of large probabilities in *γ*) or in the third quadrant (indicating consistent risk-averse behavior in *α* and consistent underweighting of large probabilities in *γ*). Such a tendency is particularly evident in utility for both body posture and elevation. Our fits suggest more idiosyncratic behavior in probability weighting between conditions, with a larger number of data points lying in the second and fourth quadrants. Fisher’s exact test did not uncover a significant difference between the number of subjects with *α* < 1 between SIT and STAND, nor between the Low and High elevation conditions for either body posture. Similarly, Fisher’s exact test did not reveal significant differences for the number of subjects with *γ* < 1. Pearson’s product-moment correlation coefficient was computed to further evaluate the relationship between conditions based on *α* and *γ*. At both elevations, there were strong positive correlations between body postures for each parameter. For both the SIT and STAND conditions, there were moderate positive correlations between elevations for *α*; for SIT there was a weak negative correlation between elevations for *γ*, and for STAND there was a weak positive correlation between elevations for *γ*. Permutation tests did not reveal significant differences in either parameter between postures or elevations.

**Table 1:** Median CPT parameter fits.

	*α*	*γ*	*k*
SIT Low	0.52 [0.20, 1.38]	0.91 [0.39, 1.22]	8.37 [0, 12.08]
STAND Low	0.68 [0.28, 1.23]	0.90 [0.52, 1.24]	6.88 [1.52, 18.21]
SIT High	0.67 [0.20, 1.51]	0.79 [0.62, 1.07]	6.75 [0.90, 21.01]
STAND High	0.37 [0.25, 1.40]	0.72 [0.45, 1.34]	9.74 [2.59, 29.39]

### Comparison to previous findings

Ultimately, the postures and postural threat presented in this experiment did not affect economic decision making in healthy young adults. Our findings indicate that neutral postures such as sitting and standing are inconsequential to an unrelated economic task, and risk-sensitivity in an economic domain is less sensitive to emotional state than in the motor domain.

Previous studies have used similar lottery paradigms to investigate risk-sensitivity in economic tasks. Wu, Delgado & Maloney (2009) and Wu, Delgado & Maloney (2011) analyzed subject choices across economic lotteries and equivalent motor lotteries for a rapid pointing task. Their resulting median parameters for the economic task align with our findings, suggesting risk-aversion in utility and underweighting of large probabilities. Jarvstad et al. (2013) found comparable trends in utility and probability weighting for an economic lottery series using best fits from eight parameterizations of the CPT model.

We expected that subjects would become more risk-averse with more difficult postures and with increased postural threat. The different conditions presented in this experiment—standing compared to sitting and atop the 0.8 m platform compared to ground level—are intended to elicit feelings of discomfort, instability, and fear of falling. Elevation in particular has a notable affect on psycho-social measures. Anxiety and fear of falling increase at the edge of a real or virtual elevated platform, while perceived confidence and stability decrease (Adkin et al., 2002; Cleworth, Horslen & Carpenter, 2012). These state changes are thought to induce altered performance in motor control tasks at increased surface heights due to the selection of a cautious strategy by the CNS. Specifically, individuals adopt a stiffening strategy and exhibit a limited range of motion at the edge of an elevated platform compared to ground level. Carpenter et al. (2001) showed this was true at a height of 0.81 m, which is approximately the same height as the elevation threat presented in our experiment. We reasoned that a cautious policy, resulting from emotional feelings of threat and manifesting itself in the motor domain (which is highly salient to postural threat due to the potential for a fall), could also influence risk-sensitivity in an unrelated economic task.

Concerning our examination of postures, we have described that standing is less comfortable, more unstable, and more cognitively loading than sitting. Cognitive loading has been shown to alter risk preferences, as Whitney, Rinehart & Hinson (2008) demonstrated in a dual-task study of working memory and economic decisions under risk. When asked to memorize a string of alphabetic letters prior to choosing between a sure gain or loss and a gamble, subjects chose the gamble less often than when they did not receive a prior cognitive loading. This behavior was attributed to subjects choosing the computationally simple option due to a limited ability to process risk under the cognitive load. In our experiment, such behavior would lead to more risk-averse tendencies under the additional cognitive load of standing compared to sitting. Alternatively, it has been shown that we are more likely to choose options that have higher affective impact when cognitive resources are engaged in other tasks (Shiv & Fedorikhin, 1999). This suggests that high-reward lotteries would be favored more during a task with higher cognitive load, which would lead to more risk-seeking tendencies during standing compared to sitting. Our findings were inconsistent with either of the above reasonings, as risk preferences definitively did not change between sitting and standing. Indeed, the increased cognitive load during standing may not be large enough to trigger significant changes in risk-sensitivity. The additional attention required during standing does not necessarily deplete cognitive processes required for higher-level decisions. For example, in a study of potential effects of workplace posture for airport security screeners, there was no difference in screening performance between sitting and standing (Drury et al., 2008). Similar risk preferences between sitting and standing substantiate the idea that risk-sensitivity in a movement domain, as in O’Brien & Ahmed (2013), is indeed a result of the actual movements and not simply due to the postures assumed during testing.

Actions have previously been shown to alter our perceptions (Witt & Brockmole, 2012). In our experiment, any action-related implications of a standing posture or increased elevation did not appear to affect perceptions of risk, either in utility or interpretations of probability. Despite feeling more threatened at a high elevation, as seen in skin conductance measures, our subjects’ choices did not reflect an altered perception of the lottery risks. It is possible, however, that our elevated platform was not high enough to influence risk preferences. Other studies of elevation continue to see large changes in motor behavior at heights greater than 1.5 m (Adkin et al., 2000; Adkin et al., 2002; Adkin et al., 2008; Davis et al., 2009; Cleworth, Horslen & Carpenter, 2012). Although elevations of ∼0.8 m do induce cautious motor strategies, conjunctive effects in an economic task may be muted at such a height, perhaps because the threat is less salient to this task. Although the platform height in this experiment was constrained by our laboratory ceiling, we are pursuing alternative techniques to increase perceived threat.

## Conclusions

This is the first study to examine the effect of posture or postural threat on economic decision-making. In this experiment, we compared risk-sensitivity between four conditions, including manipulations of body posture (sitting vs. standing) and threat (low elevation vs. high elevation). We recorded subjects’ choices in a series of two-alternative economic lotteries, and we fit these choices to a model based on cumulative prospect theory. Neither altered body posture nor increased postural threat affected risk-sensitivity. Skin conductance, a measure of physiological arousal, did not change with body posture but did increase with elevation, confirming that the protocol was sensitive enough to discriminate between conditions. We conclude that economic choices possess a degree of robustness relative to emotional state, remaining relatively consistent in the presence of modest postural threat.

## Supplemental Information

10.7717/peerj.475/supp-1 Data S1 Lottery and choice data (all subjects) Individual folders and data files for each of the 13 subjects who participated in the experiment. There are four files within each subject’s folder: one for each financial (FIN) task, including SIT Low, SIT High, STAND Low, and STAND High. The files are named as follows: S_subject ID_FINelevation_posture_trialchoices.dat
Each file contains the lottery information presented to the subject on each trial, ordered from Trial 1 through Trial 72, as well as the subject’s choice on that trial. Specifically, the columns in each data file are: probability for Lottery A, reward for Lottery A, probability for Lottery B, reward for Lottery B, and trial choice (A, B, or NaN for no response).
